# Recommendations to improve pharmacy practice research in the Middle Eastern Arab countries

**DOI:** 10.1186/s40545-021-00357-0

**Published:** 2021-08-20

**Authors:** Daneh Obaid, Faris El-Dahiyat, Zaheer-Ud-Din Babar

**Affiliations:** 1 College of Pharmacy, Al Ain University, P.O Box 64141, Al Ain, UAE; 2 Department of Pharmacy, School of Applied Sciences, University of Huddersfield, Huddersfield, HD1 3DH West Yorkshire UK

**Keywords:** Pharmacy practice research, Evidence-based practice, Middle East, Arab countries, Recommendation

## Abstract

The expansion of the pharmacist-led services has resulted in increased demand to have evidence in terms of necessity, efficacy, and cost. Evidence-based practice is crucial to provide the best patient health outcomes and pharmacy practice research can play a key role in this regard. This commentary provides a background of pharmacy practice research, and then, it highlights three key recommendations based on a systematic review of the literature. The systematic review of the literature on pharmacy practice research has been conducted in 12 Middle Eastern Arab Countries. The three key recommendations include (a) identification of research priorities by health authorities in each country; (b) pharmacy practice research culture to be nurtured and promoted with academic researchers and practitioners; and (c) shifting pharmacy practice research towards applied, interventional, and implementation studies

## Introduction

Pharmacy practice research can generate evidence to inform policymakers. It also conveys the value for potential new roles and services [1, 2]. There is a varied definition of pharmacy practice or pharmacy practice research [3]. The FIP definition states pharmacy practice research as a “component of health services research that emphasizes the impact of the practice of pharmacy on the healthcare systems, medicines use, and patient care [4]. Its scope has expanded over the past few decades to encompass aspects such as the clinical, behavioural, economic and humanistic implications of the pharmacy practice as well as practice change and implementation of new services in routine practice” [4].

Pharmacy practice is an important component in the health system of high-income countries [5]. However, to improve medicines use, practice research has proven vital and it is a key in the countries with developing health systems [6]. Pharmacy education and pharmacy practice in the Arab Middle Eastern countries continues to evolve. Currently, there is no published comprehensive review that outlines the overall status of pharmacy practice research in this region. Hence, a review was conducted recently to gauge the status of pharmacy practice research published literature in the 12 Arabic-speaking Middle Eastern Countries [7].

Medline/PubMed and Scopus were used to screen and retrieve the articles. The articles published in the English language related to any aspect of pharmacy practice in 12 countries Bahrain, Iraq, Kingdom of Saudi Arabia, Kuwait, Lebanon, Oman, Palestine, Qatar, Syria, United Arab Emirates, Yemen, and Jordan during 2009–2019 were included.

Seven themes came out from these selected articles. Medication use was the predominant 30.78% (302) theme, followed by pharmacy practice and pharmacist services 22.73% (223), pharmacy education 16.61% (163), medication safety and pharmacovigilance 13.56% (133), pharmacoeconomics and pharmaceutical marketing 8.22% (77), clinical research 6.73% (66), medicines information, and public health promotion1.81% (17). The Kingdom of Saudi Arabia (KSA), Jordan, Qatar, and the UAE were the leading countries to publish research. Studies are scarce regarding the implementation of pharmacy services. Following is a key set of recommendations based on these findings [7].

## Recommendations to improve pharmacy practice research in the Middle East

### Identification of research priorities by health authorities in each country

Planning and setting an agenda for pharmacy practice research are needed. This agenda-setting needs to be done by the Ministries of Health in these countries. It will maximize the value of the studies and will also help bridge the gaps in the pharmacy practice research. This can also help in avoiding the repetition of ideas and resources. Girolamo and Reynders (2020) and Dawoud, D. et. Al., also emphasized the need to have a pharmacy practice research priorities related to the COVID-19 pandemic [8, 9].

### Pharmacy practice research culture to be nurtured and promoted with academic researchers and practitioners

Universities and academic institutions are proposed to take the lead and creating a national health research hub. A network of researchers can help build large-scale studies. The combination of knowledge, skills, and resources that will be accessible in the research networks collectively will promote the research outcomes with valid and reliable results. This practice had a remarkable influence in several developed countries, and therefore, it is suggested that a similar approach could be used in the Middle East countries too [10–12]. Currently, it was observed that a large number of smaller studies are being conducted. It is hoped that international collaborations will facilitate conducting large-scale projects [13].

### Shifting pharmacy practice research towards applied, interventional, and implementation studies

In the recent review of the literature, it was observed that there was a lack of interventional studies assessing pharmacy services. The literature was also scant on the development of new services [7]. Pharmacy practice research has the potential to develop interventions by evaluating practice requirements, and also by assessing the effectiveness of these interventions.

A health system can only be strengthened by applying evidence-based practice hence robust studies are needed which are large scale, and of applied, interventional nature showing multidisciplinary and cross-disciplinary collaboration.

## Data Availability

Not applicable.
